# KRAS-ERK signaling drives metastasis in colorectal cancer via phosphorylation-dependent activation of the ZBTB20-TGFBR2 axis

**DOI:** 10.1186/s13046-025-03619-w

**Published:** 2026-01-02

**Authors:** Qincheng Liu, Jieru Huang, Zhe Zhang, Zhijian Xu, Shanshan Li, Wu Guo, Xi Liu, Tao Shen, Silvia Vega-Rubín-de-Celis, Qiang Li, Runya Fang, Yongjie Wei

**Affiliations:** 1 School of Medicine, Affiliated Shanghai East Hospital, Tongji University, Shanghai, China; 2 School of Pharmaceutical Science, Fudan University, Shanghai, China; 3 Guangzhou Institution of Cancer Research & the Affiliated Cancer Hospital, Guangzhou Medical University, Guangzhou, China; 4 School of Life Science, Guangzhou Medical University, Guangzhou, China; 5 Department of Pathology, The First Affiliated Hospital of Xi’an Jiaotong University, Xi’an, China; 6 College of Biotechnology and Pharmaceutical Engineering, Nanjing Tech University, Nanjing, China; 7 Institute for Cell Biology (Cancer Research), University Hospital Essen, Hufelandstrasse 55, Essen, 45147 Germany

**Keywords:** Colorectal Cancer, KRAS Mutation, Metastasis, ZBTB20, ERK, Phosphorylation, Drug Resistance

## Abstract

**Background:**

Metastatic colorectal cancer (CRC) harboring KRAS mutations presents a major therapeutic challenge due to its aggressive nature, poor prognosis, and resistance to EGFR-targeted therapies. This study aimed to identify novel drivers of metastasis specifically in KRAS-mutant CRC and to elucidate the underlying molecular mechanisms to undercover new therapeutic vulnerabilities.

**Methods:**

We integrated data from clinical databases (TCGA, CPTAC) with experimental validation using human CRC cell lines, a tissue microarray, and two distinct in vivo metastasis models (liver and lung colonization). ZBTB20 expression and function were analyzed by IHC, Western blotting, Transwell assays, and RNA-seq integrated with ChIP-seq data. The mechanism of ZBTB20 regulation was investigated via co-immunoprecipitation, mass spectrometry, truncation analysis, site-directed mutagenesis, and luciferase reporter assays. Statistical significance was determined using Student’s t-tests, ANOVA, and survival analysis.

**Results:**

ZBTB20 expression was significantly upregulated with metastatic progression specifically in KRAS-mutant CRC patients and correlated with reduced overall survival. Functionally, ZBTB20 promoted CRC cell migration, invasion, EMT in vitro, and drove metastatic colonization in vivo. Mechanistically, KRAS/ERK signaling directly phosphorylated ZBTB20 at Threonine 138, 142, and 232, a step essential for its nuclear localization and pro-metastatic activity. Integrating transcriptomic and cistromic data, we identified TGFBR2 as a direct transcriptional target of activated ZBTB20. Notably, pharmacological degradation of TGFBR2 with the inhibitor ITD-1 potently abrogated metastatic outgrowth in both liver and lung colonization models.

**Conclusions:**

Our findings delineate a novel KRAS-ERK-ZBTB20-TGFBR2 signaling axis that is a critical driver of metastasis colonization in KRAS-mutant CRC. The robust efficacy of a TGFBR2 degrader in multiple in vivo models validates this axis as a viable therapeutic target, offering a promising strategy to inhibit metastatic progression in patients with this aggressive disease.

**Graphical Abstract:**

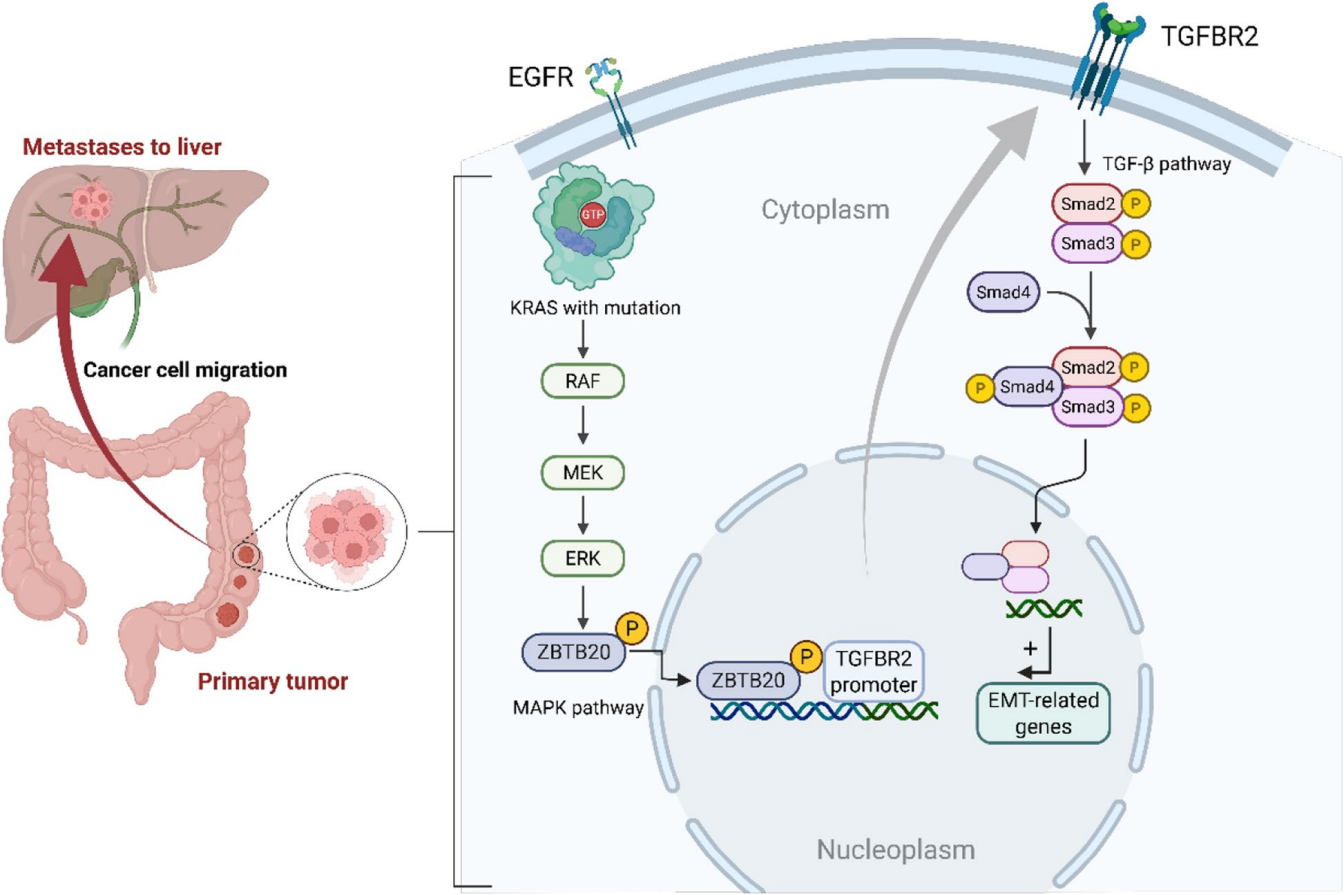

**Supplementary Information:**

The online version contains supplementary material available at 10.1186/s13046-025-03619-w.

## Introduction

Colorectal cancer (CRC) represents a substantial global health burden, ranking as a leading cause of cancer-related incidence and mortality worldwide [1]. While localized CRC offers a favorable prognosis, metastatic disease, particularly liver metastasis which is common due to colorectal venous drainage patterns, results in drastically reduced survival rates, dropping below 15% at 5 years [2, 3]. The development of CRC follows a multi-step progression involving the accumulation of genetic alterations, famously termed the “adenoma-carcinoma sequence,” often initiated by *APC* mutations [4] and subsequently driven by mutations in genes like *KRAS*, *SMAD4*, and *TP53 *[5].

Activating mutations in the *KRAS* oncogene, found in approximately 40% of CRCs, are pivotal drivers of tumorigenesis and therapeutic resistance [6]. These mutations, typically at codons 12, 13, or 61, lead to constitutive activation of KRAS signaling, predominantly through the MAPK/ERK pathway, fueling uncontrolled cell proliferation and survival [7]. Clinically, *KRAS* mutations confer resistance to anti-EGFR therapies like cetuximab and panitumumab, standard treatments for metastatic CRC, leaving a significant unmet need for effective therapies in this large patient subgroup [8, 9]. While the recent advent of KRAS G12C inhibitors like sotorasib marks a breakthrough, their efficacy is limited to a specific mutation subset, and responses in CRC have been modest, highlighting the urgent need for alternative strategies targeting KRAS-driven pathology [10, 11] common G12D/V mutations, especially for the more common G12D/V mutations [12–15]. Furthermore, KRAS mutations are independent prognostic factors associated with more aggressive disease and shorter patient survival [16], often correlating with specific molecular subtypes like CMS3 [17, 18].

Metastasis, the primary cause of CRC mortality, involves complex signaling networks that enable cancer cells to invade, disseminate, and colonize distant organs [19]. Identifying key nodes within these networks, particularly those downstream of intractable oncogenes like mutant KRAS, is crucial for developing new anti-metastatic therapies. A central challenge, therefore, is to pinpoint which specific stages of this metastatic cascade are most vulnerable to therapeutic intervention. Given that transcription factors (TFs) serve as the ultimate molecular integrators of upstream signaling and executors of the metastatic gene program (e.g., EMT), in this study, we sought to identify novel TF regulators of metastatic progression specifically operative in the context of KRAS-mutant CRC. Through integrated bioinformatic analysis and experimental validation, we pinpoint the transcription factor ZBTB20 as a critical driver. We demonstrate that ZBTB20 expression increases with metastatic progression in KRAS-mutant CRC and correlates with poor outcomes. We then unravel a novel regulatory mechanism whereby oncogenic KRAS signaling, via ERK, directly phosphorylates ZBTB20, controlling its nuclear localization and transcriptional activity. Crucially, we identify *TGFBR2*, encoding a key receptor in the pro-metastatic TGF-β pathway, as a direct transcriptional target of activated ZBTB20. Our findings establish the KRAS-ERK-ZBTB20-TGFBR2 axis as a novel pathway promoting EMT and metastasis in KRAS-mutant CRC, and validate this axis as a potent and broadly effective therapeutic target for inhibiting metastasis.

## Materials and methods

### Cell lines, reagents, and constructs

The study utilized the isogenic KRAS G12V-mutant human CRC cell lines SW480 (primary) and SW620 (metastatic). Pharmacological agents included the MEK/ERK inhibitor AZD8330 and the specific TGFBR2 degrader ITD-1. The phosphorylation-deficient ZBTB20-3T/A mutant (T138A, T142A, T232A) was generated by site-directed mutagenesis to abolish ERK-mediated phosphorylation. Detailed cell culture conditions, antibody information, and stable cell line generation protocols are provided in the Supplementary Methods.

### Mechanistic and molecular analysis

#### Identification of ZBTB20 regulation and target

##### **Co-IP and mass spectrometry**

FLAG-tagged ZBTB20 was immunoprecipitated from SW480 cells, and co-precipitated proteins were analyzed by LC–MS/MS to identify MAPK1 (ERK2) as an interacting kinase.

##### **Phosphorylation and localization**

ZBTB20 phosphorylation was detected using an anti-phospho-threonine-proline (pTP) motif antibody. The requirement of phosphorylation for ZBTB20 nuclear localization was assessed by Immunofluorescence (IF) and nuclear/cytoplasmic fractionation following AZD8330 treatment.

##### Transcriptional target identification

RNA sequencing (RNA-seq) of ZBTB20-knockdown SW620 cells was integrated with publicly available ZBTB20 ChIP-seq data to identify high-confidence direct targets.

##### Direct binding assays

Direct transcriptional control of *TGFBR2* was confirmed by Luciferase Reporter Assays and Electrophoretic Mobility Shift Assays (EMSA) using recombinant ZBTB20 protein and *TGFBR2* promoter probes.

#### In vivo metastasis and therapeutic validation

All animal experiments were approved by the Institutional Animal Care and Use Committee.

#### Metastasis models

The necessity of ZBTB20 was tested in the SW620 loss-of-function model (shZBTB20). The functional requirement of ZBTB20 phosphorylation was tested in the SW480 gain-of-function model (ZBTB20-WT vs. ZBTB20-3T/A mutant). Both models utilized intrahepatic injection for liver metastasis and tail vein injection for lung colonization.

#### Pharmacological intervention

The therapeutic efficacy of the ZBTB20-TGFBR2 axis was validated by systemic administration of the TGFBR2 degrader ITD-1 (20 mg/kg, daily) in the highly metastatic SW620 liver and lung colonization models. Metastatic burden was monitored by bioluminescence imaging (BLI) and confirmed by H&E staining and Immunohistochemistry (IHC).

#### Clinical data analysis and statistics

Clinical data from the TCGA and CPTAC datasets were analyzed to establish the KRAS-specific correlation of ZBTB20 and TGFBR2 expression with metastatic progression and patient survival. Detailed statistical methods, including the use of Student’s t-tests, ANOVA, and survival analysis, are provided in the Supplementary Methods.

## Results

### ZBTB20 upregulation is specifically associated with metastatic progression in KRAS-mutant CRC and is a prognostic marker for poor survival

**Fig. 1:**
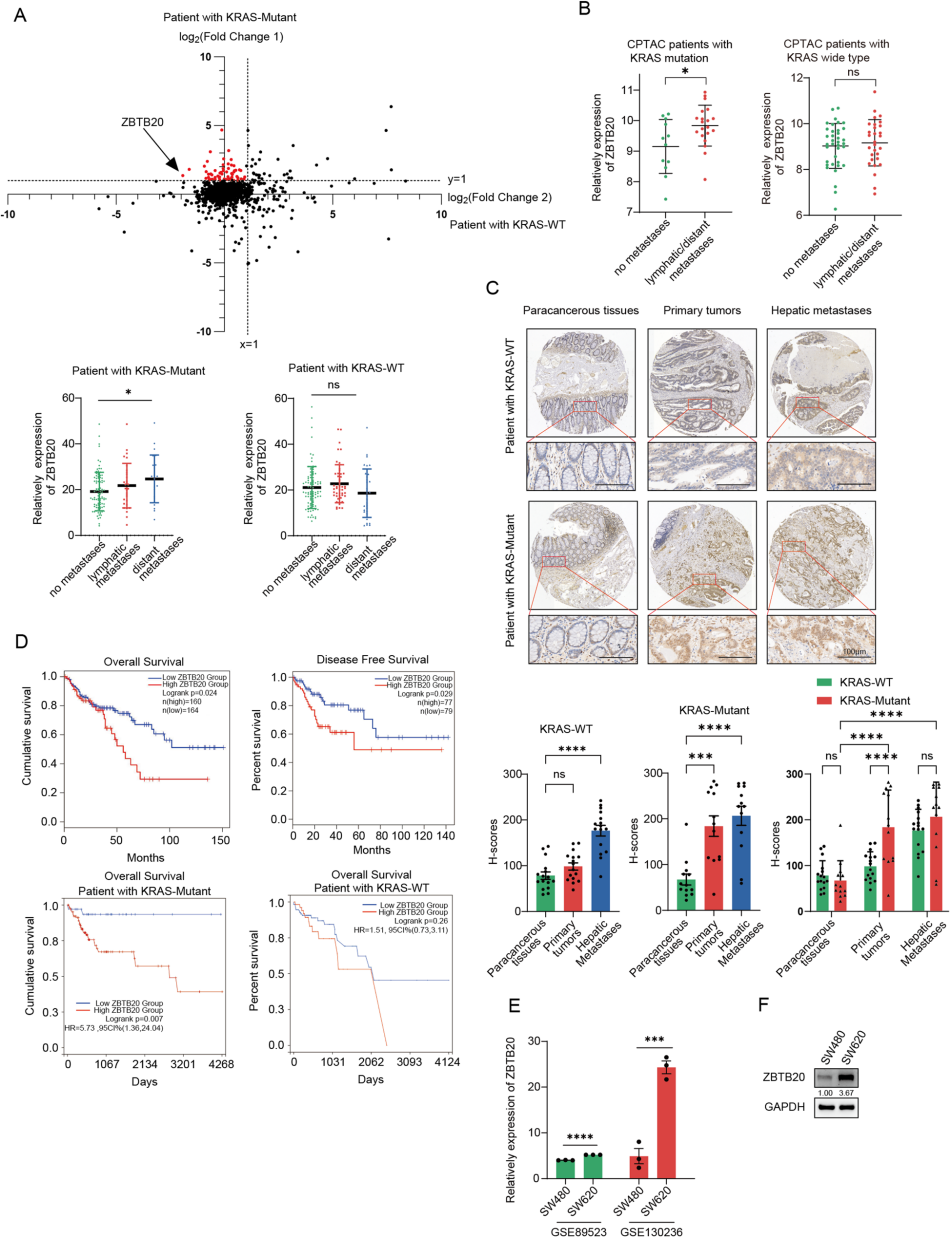
ZBTB20 expression is upregulated during metastatic progression, particularly in KRAS-mutant CRC, and correlates with poor prognosis. **A** Top: Scatter plot from TCGA data comparing the log2(Fold Change) of gene expression (metastatic vs. non-metastatic) in KRAS-mutant (Y-axis) versus KRAS-WT (X-axis) CRC patients. Only genes annotated as transcription factors are shown (red dots). ZBTB20 is highlighted by an arrow as a top candidate, selected based on its distinct expression pattern: a strong upregulation in the KRAS-mutant cohort (high log2 Fold Change on the Y-axis) concurrent with a negligible change in the KRAS-WT cohort (log2 Fold Change near zero on the X-axis). Bottom: Relative expression of ZBTB20 mRNA stratified by metastatic status in patients with KRAS-mutant (left) and KRAS-WT (right) tumors. **B** Relative expression of ZBTB20 protein from the CPTAC dataset in non-metastatic vs. metastatic tumors from patients with KRAS-mutant (left) and KRAS-WT (right) CRC. **C** Representative IHC images and corresponding quantification (H-scores) of ZBTB20 staining from a human CRC tissue (*n* = 13 KRAS-mutant, *n* = 16 KRAS-wild-type) microarray showing paracancerous tissues, primary tumors, and hepatic metastases from both KRAS-WT and KRAS-mutant patient cohorts. Note the significant upregulation of ZBTB20 in the KRAS-mutant cohort compared to the KRAS-wild-type cohort at the Primary Tumor stage, and the significant increase from Primary Tumor to Hepatic Metastases specifically within the KRAS-mutant cohort. The brackets and asterisks indicate significant differences determined by a two-way ANOVA followed by Tukey’s multiple comparisons test (****p < 0.0001; ns, not significant). **D** Kaplan–Meier analyses of patient survival from the TCGA CRC cohort, stratified by high vs. low ZBTB20 expression. Top left: Overall Survival (OS) in the entire patient cohort. Top right: Disease-Free Survival (DFS) in the entire cohort. Bottom left: OS analysis in the KRAS-mutant subgroup, demonstrating a significant association between high ZBTB20 and poor prognosis. Bottom right: OS analysis in the KRAS-wild-type subgroup, showing no significant association. *P*-values were determined by log-rank test. **E** ZBTB20 mRNA expression in the primary CRC cell line SW480 versus its isogenic metastatic counterpart SW620, analyzed from public GEO datasets. **F** Western blot analysis confirming higher ZBTB20 protein expression in SW620 cells compared to SW480 cells. GAPDH served as a loading control. Data in (**A**-**F**) are presented as mean ± SD. *p < 0.05, ***p < 0.001, ****p < 0.0001; ns, not significant. Statistical analyses for (**C**) were performed using Two-way ANOVA followed by Tukey’s post-hoc test. Statistical analyses for (**A**, **B**, **E**) were performed using one-way ANOVA or Student’s t-test. Scale bars are as indicated

To identify genes specifically associated with metastatic progression in KRAS-mutant CRC, we first compared the transcriptomic fold-changes (metastatic vs. non-metastatic) between KRAS-mutant and KRAS-wild-type (WT) patient cohorts from the TCGA database. Our selection criteria were designed to pinpoint a driver with a distinct differential pattern: 1) a significant upregulation with metastasis in the KRAS-mutant cohort, and 2) a minimal or non-significant change in the KRAS-WT cohort. Among the candidates, ZBTB20 emerged as a top candidate because it most robustly fulfilled these criteria, demonstrating a substantially greater increase in expression with metastasis specifically in the KRAS-mutant context (Fig. 1A, top panel). Quantitative analysis confirmed that ZBTB20 mRNA expression significantly increased with metastatic stage only in the KRAS-mutant cohort (Fig. 1A, bottom panel). This KRAS-specific association was further corroborated at the protein level using the CPTAC dataset, which also showed a significant elevation of ZBTB20 protein in metastatic versus non-metastatic tumors from patients with KRAS mutations (Fig. 1B).

To validate this crucial KRAS-specific association at the protein level, we performed immunohistochemistry (IHC) on a commercial CRC tissue microarray, analyzing a curated cohort of 13 KRAS-mutant and 16 KRAS-wild-type patient samples. The results provided striking confirmation of our hypothesis. In the KRAS-mutant cohort, ZBTB20 protein levels showed a significant, stepwise increase at each stage of progression, rising from paracancerous tissue to primary tumors, and again to the highest levels in hepatic metastases. Crucially, the increase in ZBTB20 expression during the progression from primary tumor to hepatic metastasis was statistically significant (Fig. 1C, bottom panel). In stark contrast, the KRAS-WT cohort lacked this clear stepwise progression. Most notably, while there was an overall increase from paracancerous to metastatic tissue, there was no significant change in ZBTB20 expression during the critical transition from primary tumor to hepatic metastasis (Fig. 1C, top panel). This demonstrates that the progressive, stepwise upregulation of ZBTB20 is a hallmark of metastatic progression exclusive to KRAS-mutant CRC.

Consistent with its association with aggressive disease, high ZBTB20 expression emerged as a significant predictor of worse overall survival in the broader CRC patient population (Fig. 1D, top left, Log-rank *p* = 0.024). Crucially, when we stratified the analysis by KRAS mutation status, we found that this prognostic significance was exclusively driven by the KRAS-mutant cohort. High ZBTB20 expression was strongly associated with poorer overall survival in patients with KRAS-mutant tumors (*p* = 0.007, Fig. 1D, bottom left), but this association was completely lost in the KRAS-wild-type cohort (*p* = 0.26, Fig. 1D, bottom right). Furthermore, high ZBTB20 expression also correlated significantly with worse disease-free survival (*p* = 0.029, Fig. 1D, top right), further highlighting its role in disease progression and tumor recurrence.). Finally, this clinical observation was mirrored in our in vitro model system, where both mRNA and protein levels of ZBTB20 were significantly higher in the metastatic KRAS-mutant SW620 cell line compared to its isogenic primary counterpart, SW480 (Fig. 1E, F).

Collectively, these data from multiple patient cohorts and analytical platforms identify ZBTB20 upregulation as a hallmark of metastatic progression exclusive to KRAS-mutant CRC. Guided by this strong clinical evidence pointing to a specific pathogenic role in this context, we proceeded to investigate its functional consequences in relevant KRAS-mutant cellular models.

### ZBTB20 promotes CRC metastatic phenotypes in vitro and drives liver metastasis in vivo

**Fig. 2:**
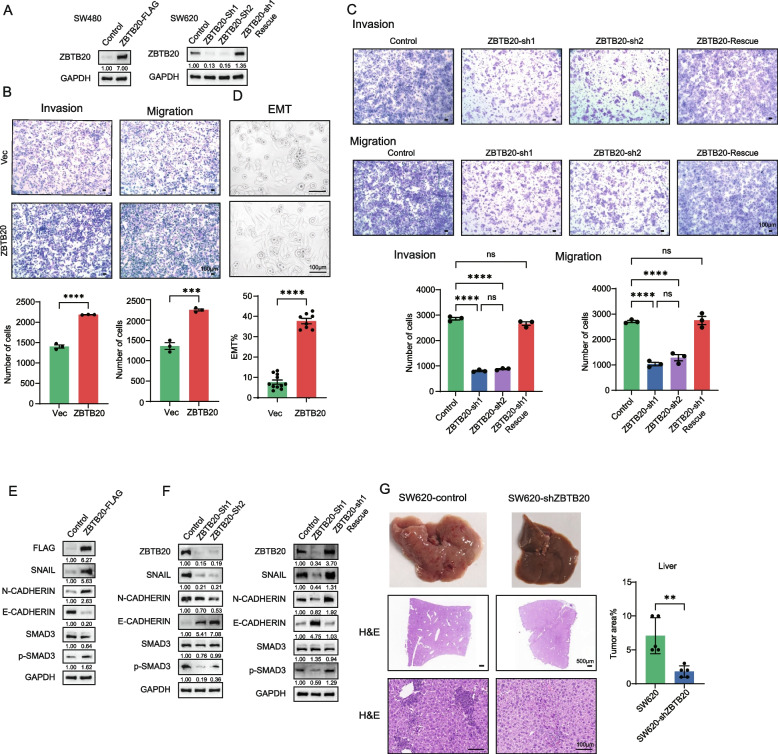
ZBTB20 promotes metastatic phenotypes in vitro and drives liver metastasis in vivo. **A** Western blot analysis confirming stable ZBTB20 overexpression in SW480 cells knockdown in SW620 cells, and re-expression in the SW620-Rescue cell line. **B** Transwell migration and invasion assays of SW480-Vec and SW480-ZBTB20 cells. **C** Transwell migration and invasion assays of SW620-Control and SW620-shZBTB20 cells. **D** Morphological analysis showing ZBTB20-induced EMT in SW480 cells. **E** Western blot analysis of EMT markers in SW480 cells following ZBTB20 overexpression. **F** Western blot analysis demonstrating that ZBTB20 knockdown alters EMT marker expression in SW620 cells (left panel), and this alteration is reversed upon ZBTB20 re-expression (right panel). **G** SW620-scramble or SW620-shZBTB20 cells (3 × 10^6) were injected into the left liver lobe of male BALB/c nude mice (*n* = 5 mice per group). Representative images of whole livers, H&E stained sections, and quantification of tumor area are shown. Data are presented as mean ± SD. *p < 0.05, ***p < 0.001, ****p < 0.0001. Scale bars are as indicated

Given the clinical correlation between ZBTB20 expression and metastasis, specifically within the KRAS-mutant subtype, we next investigated its functional role using the isogenic KRAS-mutant SW480 and SW620 cell lines, which represent an ideal system to model this disease context. In our subsequent in vitro and in vivo functional assays, the less aggressive SW480 cell line is utilized as a robust ‘gain-of-function’ model to investigate the necessary molecular *switches* (e.g., phosphorylation dependence and downstream signaling), while the highly metastatic SW620 line is employed as a ‘loss-of-function’ model to test the *dependency* on the ZBTB20 axis and the efficacy of therapeutic agents. To investigate its functional role, we generated stable SW480 cells overexpressing ZBTB20, stable SW620 cells with ZBTB20 knockdown via two independent shRNAs, and a corresponding rescue cell line by re-expressing an shRNA-resistant ZBTB20 in the knockdown cells. The modifications were confirmed by Western blot (Fig. 2A).

In the less aggressive, primary tumor-derived SW480 cell line, ZBTB20 overexpression dramatically enhanced migratory and invasive capabilities (Fig. 2B) and induced a striking morphological shift characteristic of epithelial-mesenchymal transition (EMT) (Fig. 2D). At the molecular level, this was accompanied by decreased expression of the epithelial marker E-cadherin and increased expression of the mesenchymal markers SNAIL, ZEB1, and N-cadherin (Fig. 2E).

Conversely, in the highly metastatic SW620 cell line, knockdown of ZBTB20 significantly impaired both migration and invasion (Fig. 2C). To determine if these pro-metastatic functions were independent of cell proliferation, we performed CCK-8 and colony formation assays. Neither ZBTB20 overexpression in SW480 cells nor its knockdown in SW620 cells had a significant effect on cell proliferation rates or colony-forming capabilities (Supplemental Figure S1A-D). These findings strongly suggest that ZBTB20’s primary function in KRAS-mutant CRC is to specifically promote cell motility and invasion, rather than regulating cell growth. This functional impairment was associated with a molecular reversal of the mesenchymal phenotype, evidenced by increased E-cadherin and decreased SNAIL and N-cadherin expression (Fig. 2F, left panel). Crucially, both the migratory defects and the altered EMT marker expression were fully restored upon re-expression of ZBTB20 in a rescue cell line, confirming the specificity of these effects (Fig. 2C and F, right panel).

To validate these findings and directly test the necessity of ZBTB20 for metastatic progression in vivo, we first utilized the highly metastatic SW620 cell line in an orthotopic liver metastasis model. This well-established metastatic model provides a robust and sensitive system to assess the impact of ZBTB20 loss-of-function. Mice injected with ZBTB20-knockdown cells exhibited a dramatic reduction in liver metastatic burden compared to controls (Fig. 2G). This was evident from the gross morphology of the livers and confirmed by histological analysis and quantification of tumor area.

Collectively, these in vitro and in vivo data demonstrate that ZBTB20 is a potent driver of metastatic phenotypes in CRC, promoting cell motility, EMT, and the colonization and subsequent outgrowth of tumor cells in distant organs.

### ZBTB20 is a direct substrate of ERK1/2 and is phosphorylated at threonine 138, 142, and 232

**Fig. 3:**
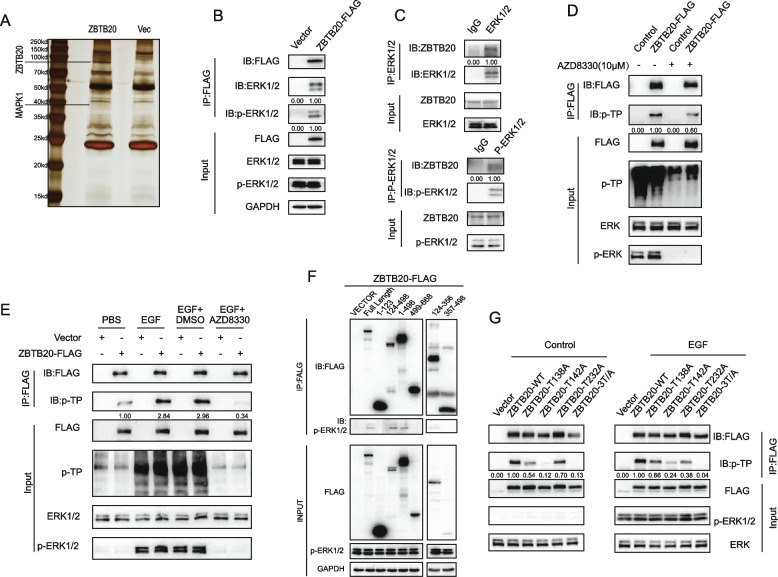
ZBTB20 interacts with and is directly phosphorylated by ERK1/2 at Threonine 138, 142, and 232. **A** Silver stain of proteins co-immunoprecipitated with ZBTB20-FLAG from SW480 cells, with the MAPK1 (ERK2) band identified by mass spectrometry indicated. **B**, **C** Co-immunoprecipitation (Co-IP) in SW480 cells showing the interaction between ZBTB20 and total/phosphorylated ERK1/2, confirmed by both forward (**B**) and reverse (**C**) IPs. **D** ZBTB20 phosphorylation in SW480 cells is MEK/ERK-dependent, as shown by treatment with the inhibitor AZD8330. Phosphorylation was detected using an anti-phospho-threonine-proline (pTP) motif antibody. **E** EGF-induced phosphorylation of ZBTB20 is dependent on the MEK/ERK signaling pathway. HEK293T cells expressing ZBTB20-FLAG were serum-starved and then stimulated with EGF in the presence or absence of the MEK/ERK inhibitor AZD8330. Phosphorylation was detected by immunoprecipitation of ZBTB20 followed by immunoblotting with an anti-phospho-threonine-proline (pTP) motif antibody. **F**, **G** Mapping and validation of ERK phosphorylation sites in HEK293T cells. Cells expressing the indicated ZBTB20 constructs were serum-starved and stimulated with EGF. Immunoprecipitated proteins were then analyzed by immunoblotting. **F** Truncation analysis identified the 124–356 amino acid region as the primary ERK-interaction domain. **G** Site-directed mutagenesis of threonine residues within this domain identified T138, T142, and T232 as the bona fide phosphorylation sites, with phosphorylation detected using the anti-pTP antibody. Scale bars are as indicated. Quantification ratios above the bands in (**D**, **E**, **G**) represent the relative pTP signal normalized to total ZBTB20 (IB:FLAG)

Given the correlation between ZBTB20 expression and KRAS mutation status, we hypothesized that the KRAS-MAPK signaling pathway might directly regulate ZBTB20 activity. To identify potential interacting proteins, we performed immunoprecipitation of FLAG-tagged ZBTB20 from SW480 cells followed by mass spectrometry, which identified MAPK1 (ERK2) as a prominent co-precipitating protein (Fig. 3A). We confirmed this interaction in CRC cells through co-immunoprecipitation (Co-IP) assays, demonstrating that ZBTB20 associates with the activated, phosphorylated form of ERK1/2 (Fig. 3B, C).

This interaction suggested that ZBTB20 might be an ERK substrate. Indeed, ZBTB20 immunoprecipitated from SW480 cells showed a strong basal phosphorylation signal on a phospho-threonine-proline (pTP) motif blot, which was abolished by the MEK/ERK inhibitor AZD8330 (Fig. 3D). To precisely validate this mechanism in a controlled system, we utilized HEK293T cells, which have low basal ERK activity. EGF stimulation robustly induced ZBTB20 phosphorylation. Crucially, this EGF-induced phosphorylation was completely abrogated by co-treatment with the MEK/ERK inhibitor AZD8330. This experiment provides direct evidence that ZBTB20 phosphorylation is a specific downstream event strictly dependent on MAPK/ERK pathway activation (Fig. 3E).

To map the specific region of ZBTB20 responsible for this interaction, we expressed various truncated ZBTB20 fragments in HEK293T cells and assessed their ability to bind endogenous ERK upon EGF stimulation. We found that a fragment spanning amino acids 124–356 was necessary and sufficient to co-precipitate p-ERK1/2, thus identifying this as the primary ERK-interaction domain (Fig. 3F).

Bioinformatic analysis of this 124–356 region revealed three potential S/T-P consensus motifs for ERK phosphorylation: Threonine 138 (T138), T142, and T232. To determine if these were the bona fide phosphorylation sites, we generated single and combined threonine-to-alanine (T/A) mutants and tested their phosphorylation in the inducible HEK293T system. While single mutants showed a partial reduction in EGF-induced phosphorylation, the triple mutant (T138A-T142A-T232A) completely abolished the phosphorylation signal (Fig. 3G).

These data collectively establish ZBTB20 as a direct substrate of the ERK1/2 kinase, which interacts with its 124–356 domain and phosphorylates it at T138, T142, and T232 in response to upstream signaling.

### ERK-mediated phosphorylation is essential for ZBTB20 nuclear localization and pro-metastatic function

Phosphorylation often regulates the subcellular localization of transcription factors. To investigate if this holds true for ZBTB20, we first used the inducible HEK293T cell system. Immunofluorescence analysis showed that while wild-type (WT) ZBTB20 was predominantly cytoplasmic in serum-starved cells, it translocated to the nucleus upon EGF stimulation. This nuclear entry was blocked by the MEK/ERK inhibitor AZD8330. In stark contrast, the phosphorylation-deficient triple mutant (ZBTB20-3T/A) remained confined to the cytoplasm regardless of EGF treatment, demonstrating that phosphorylation is required for its nuclear import (Supplemental Figure S2A).

**Fig. 4:**
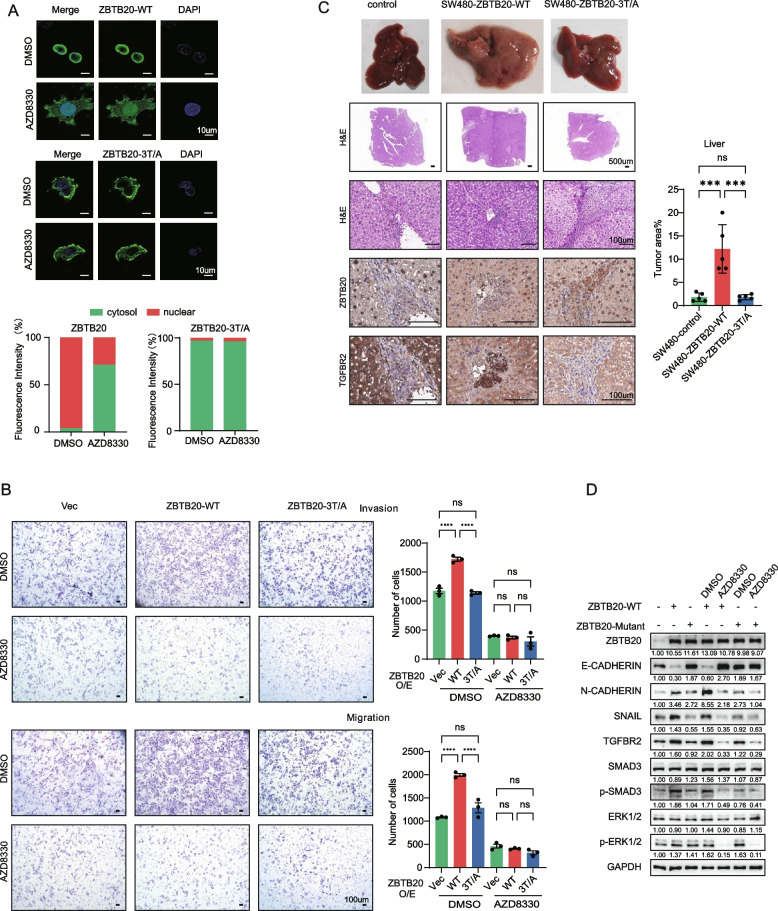
ZBTB20 phosphorylation is required for its pro-invasive function in KRAS-mutant CRC. **A** Immunofluorescence analysis of ZBTB20 subcellular localization in SW480 cells stably expressing WT ZBTB20 or the phosphorylation-deficient ZBTB20-3T/A mutant. Cells were treated with DMSO or the MEK/ERK inhibitor AZD8330 (10 µM). Green: ZBTB20; Blue: DAPI (nuclei). Quantification of nuclear vs. cytoplasmic fluorescence is shown on the right. **B** Transwell migration and invasion assays of SW480 cells expressing vector, WT ZBTB20, or ZBTB20-3T/A. Cells were treated with DMSO or AZD8330. Representative images are shown with quantification below. **C** ZBTB20 phosphorylation is required for its pro-metastatic function in vivo. SW480 stable cell lines (Vector, WT ZBTB20, or ZBTB20-3T/A; 3 × 10^6 cells per mouse) were injected into the liver lobes of male BALB/c nude mice (*n* = 5 mice per group). Representative images of whole livers, H&E stained sections, and IHC for ZBTB20 and TGFBR2 are shown. Quantification of tumor area percentage is shown in the bar graph. **D** Western blot analysis of EMT markers and downstream signaling molecules in SW480 cells stably expressing vector control, WT ZBTB20, or the phosphorylation-deficient ZBTB20-3T/A mutant. Cells were treated with DMSO or the MEK/ERK inhibitor AZD8330 (10 μM) as indicated. Data are presented as mean ± SD. *p < 0.05, ***p < 0.001, ****p < 0.0001; ns, not significant. Statistical analyses performed using one-way ANOVA. Scale bars are as indicated

We then confirmed this mechanism in the context of KRAS-mutant CRC. In SW480 cells, which have high basal ERK activity, WT ZBTB20 was localized to both the cytoplasm and the nucleus. Treatment with AZD8330 caused a clear redistribution of WT ZBTB20 to the cytoplasm. In contrast, the ZBTB20-3T/A mutant was already predominantly cytoplasmic and its localization was unaffected by ERK inhibition (Fig. 4A).

Next, we assessed the functional consequences of this phosphorylation-dependent localization in SW480 cells. In this isogenic pair, ZBTB20 overexpression in the less metastatic SW480 cell line is designed to phenocopy the hyper-active, high-expression status of ZBTB20 found endogenously in the metastatic SW620 line (Fig. 1E, F). In basal conditions (DMSO treatment), cells overexpressing WT ZBTB20 exhibited significantly enhanced migration and invasion compared to vector control cells. Crucially, this pro-metastatic function was dependent on the identified phosphorylation sites, as the ZBTB20-3T/A mutant failed to promote migration and invasion to the same extent (Fig. 4B, compare DMSO-treated groups). To confirm that this function was driven by upstream ERK signaling, we treated the cells with the MEK/ERK inhibitor AZD8330. As hypothesized, the inhibitor completely abrogated the pro-migratory and pro-invasive effects driven by WT ZBTB20, reducing them to control levels. In contrast, AZD8330 had no significant effect on the basal migration of vector cells or on cells expressing the already-impaired ZBTB20-3T/A mutant, demonstrating the specific dependence of ZBTB20’s function on a constitutively active KRAS-ERK axis (Fig. 4B, WT vs 3 T/A).

Finally, we evaluated the importance of ZBTB20 phosphorylation for metastasis in vivo using two distinct models. Consistent with our strategy to use the less aggressive SW480 cell line as a gain-of-function model to test the phosphorylation ‘switch,’ in an intrahepatic metastasis model, SW480 cells expressing WT ZBTB20 formed significantly more metastatic nodules throughout the liver compared to vector control cells. This effect was completely lost in cells expressing the ZBTB20-3T/A mutant (Fig. 4C). Similarly, in a tail vein injection model of hematogenous metastasis, WT ZBTB20 robustly promoted lung colonization, whereas the ZBTB20-3T/A mutant was unable to do so, phenocopying the vector control. Immunohistochemical analysis of the lung metastases confirmed that tumors formed by WT ZBTB20-expressing cells also had elevated levels of its downstream target, TGFBR2, an effect not seen in tumors from the mutant group (Supplemental Figure S2B). Consistent with these functional outcomes, Western blot analysis confirmed the molecular underpinnings of this phenotype (Fig. 4D). Overexpression of WT ZBTB20 induced an EMT signature, including decreased E-cadherin and increased N-cadherin, which was dependent on ERK signaling and ZBTB20 phosphorylation. This molecular switch also governed the upregulation of its target TGFBR2 and the activation of downstream SMAD3 signaling.

Together, these findings demonstrate that ERK-mediated phosphorylation is a critical switch that governs ZBTB20’s nuclear entry and is absolutely required for its ability to drive CRC cell invasion and metastasis in a KRAS-mutant context. The inability of the ZBTB20-3T/A mutant to promote metastasis confirms that the pro-metastatic function of the ZBTB20 protein, regardless of its expression level, is contingent upon its phosphorylation by the constitutive KRAS-ERK signaling.

### ZBTB20 directly upregulates TGFBR2 transcription to activate a pro-metastatic program

**Fig. 5:**
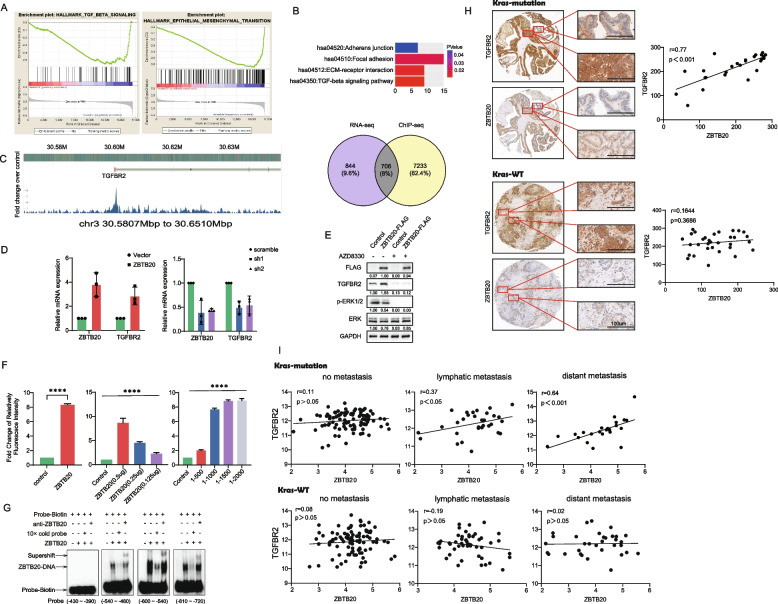
ZBTB20 directly upregulates TGFBR2 in an ERK-dependent manner, and this axis is specifically activated during metastatic progression of KRAS-mutant CRC. **A** GSEA enrichment plots showing negative enrichment of HALLMARK _TGF_BETA_SIGNALING and HALLMARK_EPITHELIAL_MESENCHYMAL _TRANSITION gene sets in SW620 cells upon ZBTB20 knockdown. **B** Venn diagram showing the overlap of differentially expressed genes from RNA-seq and genes with promoter peaks from ZBTB20 ChIP-seq data (bottom). KEGG pathway analysis of the 706 overlapping direct target genes (top). **C** Visualization of the ZBTB20 ChIP-seq binding peak at the *TGFBR2* gene locus (chr3:30.5807Mbp-30.6510Mbp). **D** qRT-PCR analysis of *ZBTB20* and *TGFBR2* mRNA levels in SW480 cells with ZBTB20 overexpression (left) and SW620 cells with ZBTB20 knockdown (right). **E** Western blot analysis showing that MEK inhibition with AZD8330 reduces TGFBR2 protein levels in ZBTB20-overexpressing SW480 cells. **F** Luciferase reporter assay showing dose-dependent activation of the *TGFBR2* promoter by ZBTB20. **G** EMSA demonstrates direct and specific binding of recombinant ZBTB20 to three distinct probes from the TGFBR2 promoter. The (−430 ~ −390) probe served as a negative control. Specificity and protein identity were confirmed by cold probe competition and an anti-ZBTB20 antibody supershift, respectively. **H** Representative IHC images and quantitative correlation analysis from a CRC tissue microarray from the same TMA cohort (as in Fig. 1C) showing co-expression and positive correlation of ZBTB20 and TGFBR2 in human KRAS-mutant CRC primary tumors and liver metastases. Scale bars indicated. **I** Correlation analysis of ZBTB20 and TGFBR2 mRNA expression in the TCGA COAD cohort, stratified by *KRAS* mutation status and metastatic stage. Pearson correlation coefficient (r) and *p*-value are shown. Scale bars are as indicated

To identify the downstream mechanisms of ZBTB20, we performed RNA-sequencing (RNA-seq) on SW620 cells following ZBTB20 knockdown (Supplemental Figure S3A). A broad pathway analysis of all differentially expressed genes revealed a significant enrichment for cancer-related and cell adhesion pathways (Supplemental Figure S3B). More specifically, Gene Set Enrichment Analysis (GSEA) highlighted a significant negative enrichment of genes associated with TGF-β signaling and Epithelial-Mesenchymal Transition (EMT), two critical pathways in metastasis (Fig. 5A, Supplemental Figure S3C).

To distinguish direct from indirect targets, we integrated our RNA-seq data with publicly available ZBTB20 ChIP-seq data. This cistromic analysis confirmed that ZBTB20 binding peaks are predominantly located within gene promoter regions (Supplemental Figure S3D) and identified 706 high-confidence direct targets (Fig. 5B). KEGG analysis of this direct-target list again confirmed a significant enrichment for the TGF-β signaling pathway, Adherens junction, and ECM-receptor interaction pathways (Fig. 5B).

Among the top candidate direct targets was *TGFBR2*, which encodes the TGF-β type II receptor. Analysis of ChIP-seq data revealed a prominent ZBTB20 binding peak within the *TGFBR2* gene locus, indicating direct physical interaction (Fig. 5C). We validated this regulatory link functionally; qRT-PCR showed that ZBTB20 overexpression increased *TGFBR2* mRNA levels, while ZBTB20 knockdown decreased them (Fig. 5D).

Importantly, we linked this transcriptional regulation to the upstream KRAS-ERK signaling axis. Treatment with the MEK inhibitor AZD8330, which blocks ERK activation, was sufficient to decrease TGFBR2 protein levels in ZBTB20-overexpressing cells (Fig. 5E), demonstrating that ERK-mediated activation of ZBTB20 is required for its ability to upregulate TGFBR2. To further confirm direct transcriptional control, luciferase reporter assays showed that ZBTB20 induced *TGFBR2* promoter activity in a dose-dependent manner (Fig. 5F). Moreover, electrophoretic mobility shift assays (EMSA) confirmed that recombinant ZBTB20 protein directly bound to specific putative binding sites within the *TGFBR2* promoter (Fig. 5G). Collectively, the integration of RNA-seq, ChIP-seq, and EMSA provides robust evidence that TGFBR2 is a bona fide direct transcriptional target of ZBTB20.

Finally, we established the clinical relevance and specificity of this axis. Immunohistochemistry (IHC) on our CRC tissue microarray revealed a strong positive correlation between ZBTB20 and TGFBR2 protein expression, particularly in KRAS-mutant tumors (*r* = 0.77, *p* < 0.001), a correlation that was absent in the KRAS-wild-type cohort (Fig. 5H). This finding was further substantiated by analysis of the TCGA COAD cohort. In KRAS-mutant patients, the positive correlation between *ZBTB20* and *TGFBR2* mRNA expression strengthened significantly with metastatic progression, peaking in patients with distant metastasis (*r* = 0.64, *p* < 0.001). Strikingly, this correlation was completely absent in KRAS wild-type patients regardless of their metastatic status (Fig. 5I).

Finally, to establish the clinical relevance of this newly identified axis, we assessed the prognostic value of TGFBR2 itself using the TCGA cohort. While high TGFBR2 expression showed a trend towards poorer overall survival (*p* = 0.09), it was a significant predictor of worse disease-free survival (*p* = 0.0063) (Supplemental Figure S4A, B). This finding indicates that elevated TGFBR2 expression, a direct downstream consequence of ZBTB20 activation, is clinically associated with a higher risk of tumor recurrence.

Together, these results identify *TGFBR2* as a direct and functionally critical transcriptional target of ZBTB20, providing a mechanistic link between KRAS-ERK signaling and the activation of a pro-metastatic program that is specifically engaged during the progression of KRAS-mutant CRC.

### TGFBR2 is a critical downstream effector of ZBTB20 and a viable therapeutic target for CRC metastasis

**Fig. 6:**
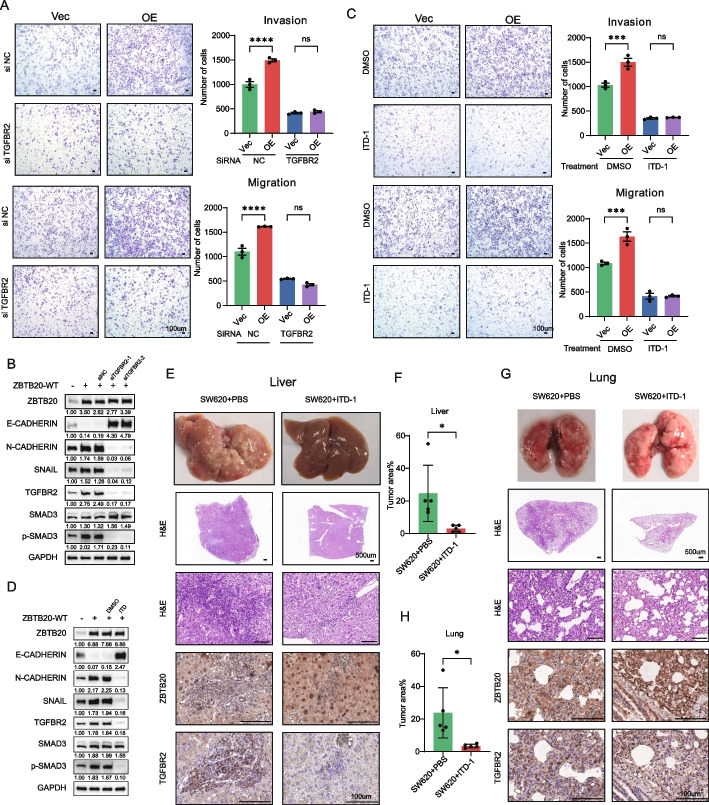
Targeted degradation of TGFBR2 abrogates ZBTB20-driven metastasis in vitro and in vivo. **A** Genetic rescue of ZBTB20-driven invasion and migration. SW480-Vec and SW480 ZBTB20 overexpression (OE) cells were treated with non-targeting control (siNC) or siRNA targeting *TGFBR2* and assessed by Transwell assays. **B** Western blot analysis confirming the molecular rescue by siTGFBR2. Knockdown of *TGFBR2* reverses the ZBTB20-induced EMT signature in SW480-OE cells. **C** Pharmacological rescue of ZBTB20-driven invasion and migration. SW480-Vec and SW480-OE cells were treated with DMSO or the TGFBR2 degrader ITD-1 and assessed by Transwell assays. **D** Western blot analysis showing that ITD-1 phenocopies the molecular rescue by reverting the ZBTB20-induced EMT signature. **E**–**H** Pharmacological degradation of TGFBR2 with ITD-1 inhibits metastatic colonization and outgrowth in vivo. For all models, male BALB/c nude mice (*n* = 5 mice per group) were treated daily with vehicle (PBS) or ITD-1 (20 mg/kg, i.p.) **E**, **F** Liver colonization model. SW620 cells (3 × 10^6) were injected intrahepatically. Representative images of whole livers, H&E sections, and IHC staining are shown (**E**), with corresponding quantification of tumor area (**F**). **G**, **H** Lung colonization model. SW620 cells (3 × 10^6) were injected via the tail vein. Representative images of whole lungs, H&E sections, and IHC staining are shown (**G**), with corresponding quantification of tumor area (**H**). Data in bar graphs are presented as mean ± SD. *p < 0.05, **p < 0.01, ***p < 0.001, ****p < 0.0001; ns, not significant. Scale bars are as indicated

Finally, we sought to determine if the transcriptional upregulation of TGFBR2 is functionally required for ZBTB20’s pro-metastatic effects and if it represents a viable therapeutic vulnerability. To test this, we used a genetic rescue strategy in our SW480 gain-of-function model. As expected, ZBTB20 overexpression (OE) significantly increased cell invasion and migration. This functional effect was completely abrogated by siRNA-mediated knockdown of *TGFBR2*, demonstrating a direct genetic dependency (Fig. 6A). We confirmed this rescue at the molecular level, where Western blot analysis showed that siTGFBR2 reversed the ZBTB20-induced EMT signature, evidenced by the restoration of E-cadherin expression (from a ZBTB20-OE ratio of 0.14 to 4.30 with siTGFBR2-1) and the suppression of mesenchymal markers N-cadherin and SNAIL (Fig. 6B). Crucially, siTGFBR2 also abrogated the accompanying increase in SMAD3 phosphorylation (p-SMAD3 ratio dropped from 2.02 to 0.23). This quantitative molecular reversal demonstrates that ZBTB20 drives EMT by functionally engaging the canonical TGF-β/SMAD pathway via its transcriptional target, TGFBR2 (Fig. 6B). To validate this pharmacologically, we utilized ITD-1, a specific inhibitor that promotes the degradation of TGFBR2 [20, 21]. Treatment with ITD-1 phenocopied the effect of siTGFBR2, potently reversing the increased invasion and migration driven by ZBTB20 overexpression (Fig. 6C). This pharmacological rescue was also confirmed at the molecular level, as ITD-1 treatment similarly reverted the ZBTB20-induced EMT signature (e.g., restoring E-cadherin from a ZBTB20-OE ratio of 0.07 to 2.47) and potently suppressed the downstream phosphorylation of SMAD3 (p-SMAD3 ratio dropped from 1.83 to 0.10), phenocopying the genetic knockdown (Fig. 6D). To further define the specificity of this pro-metastatic program, we assessed whether the ZBTB20-TGFBR2 axis also regulates cell survival. We performed apoptosis assays following the knockdown of either the upstream driver ZBTB20 or the downstream effector TGFBR2 in SW620 cells. Crucially, neither Western blot analysis for cleaved caspase-3 nor TUNEL assays showed any significant increase in apoptosis upon depletion of ZBTB20 or TGFBR2 (Supplemental Figure S5A-D). These data strongly support that ZBTB20 requires a functional TGFBR2 to execute a specific pro-metastatic program that is uncoupled from the regulation of apoptosis.

Next, we evaluated the therapeutic potential of targeting TGFBR2 in vivo. For these studies, we utilized the highly metastatic SW620 cell line, which provides a robust and consistent metastatic phenotype ideal for evaluating the efficacy of a therapeutic agent without the potential confounding variables of an exogenous overexpression system. We tested the efficacy of ITD-1 in two distinct models of metastasis: liver colonization (intrahepatic injection) and lung colonization (tail vein injection).

In the liver colonization model, systemic administration of ITD-1 significantly reduced the metastatic burden compared to vehicle-treated controls. This was evident from the gross morphology of the livers and confirmed by a marked decrease in tumor area on histological analysis (Fig. 6E, F). In the lung colonization model, ITD-1 treatment also proved highly effective, dramatically reducing the number and size of metastatic foci (Fig. 6G, H).

Immunohistochemical analysis of the residual tumors from both models confirmed the mechanism of action of ITD-1, showing a clear reduction in TGFBR2 protein levels in the inhibitor-treated groups, while ZBTB20 expression remained unchanged (Fig. 6E, F).

Taken together, these genetic and pharmacological data, validated across multiple in vitro and in vivo contexts, demonstrate that TGFBR2 is a critical downstream effector of ZBTB20 and that its targeted degradation is an effective strategy for inhibiting CRC metastasis.

## Discussion

The high frequency of KRAS mutations in CRC and their association with aggressive metastatic disease underscore the urgent need for novel therapeutic strategies. Our study addresses this challenge by identifying the transcription factor ZBTB20 as a pivotal mediator of metastasis specifically in this context. We have delineated the complete, linear signaling cascade—from oncogenic KRAS activation to ERK-mediated phosphorylation of ZBTB20, and its subsequent transcriptional upregulation of TGFBR2—that collectively drives a potent pro-metastatic program. This work provides not only a significant mechanistic insight but also validates a promising and broadly effective therapeutic strategy against the later, colonization and outgrowth stages of metastasis in this challenging patient population.

A central finding of our study is the specific dependency of ZBTB20’s pro-metastatic function on the KRAS-mutant context. Our focus was deliberately guided by compelling clinical evidence from multiple patient datasets showing that ZBTB20 upregulation is a molecular signature of metastatic progression exclusive to KRAS-mutant tumors. Furthermore, we provide a clear mechanistic basis for this specificity: ZBTB20’s pro-metastatic function requires a phosphorylation-dependent ‘activity switch’ that is flipped by the hyperactive ERK signaling inherent to KRAS-mutant tumors. We demonstrate that ERK actively phosphorylates ZBTB20 at specific threonine residues (T138, T142, T232), an event that is the critical determinant of ZBTB20’s nuclear localization and, consequently, its entire pro-metastatic capacity [22]. The specificity of this pro-metastatic program is underscored by our findings that manipulation of the ZBTB20-TGFBR2 axis affects neither cell proliferation nor apoptosis, pointing to a dedicated role in regulating cell motility and invasion.

This finding helps place our work within the broader, context-dependent roles of ZBTB20 [23] and may reconcile previous conflicting reports. The function of ZBTB20 appears to be highly plastic, as it has been reported as a tumor suppressor in other malignancies, such as hepatocellular carcinoma [24–26]. A more direct challenge arises from a previous report within colorectal cancer itself, which described ZBTB20 as an inhibitor of cell migration [26], a conclusion seemingly at odds with our findings. We propose that this apparent paradox is not a contradiction, but rather a compelling example of a transcription factor’s dual functionality, which is dictated by the specific genetic and signaling context. Our multi-layered mechanistic explanation supports this: ZBTB20’s role as a pro-metastatic driver is intrinsically linked to the KRAS-mutant status. In the absence of this oncogenic KRAS-ERK signal, ZBTB20 may be sequestered in the cytoplasm or perform a distinct, suppressive function.

This mechanism is also fundamentally distinct from the pathobiology of ZBTB20 in the developmental disorder Primrose syndrome, which is caused by loss-of-function mutations within its DNA-binding domains [27, 28]. In contrast, the phosphorylation sites we identified are located near the BTB/POZ domain and regulate ZBTB20’s subcellular localization and activity, rather than its intrinsic ability to bind DNA. Taken together, our work repositions ZBTB20 not as a static oncogene or tumor suppressor, but as a conditional pro-metastatic factor whose oncogenic potential in CRC is specifically unleashed by KRAS-ERK signaling.

Furthermore, our identification of TGFBR2 as a direct transcriptional target of ZBTB20 provides a crucial link to a well-established pro-metastatic pathway. The TGF-β pathway is known for its paradoxical role, shifting from a tumor suppressor in early lesions to a powerful promoter of invasion and immunosuppression in advanced cancers [29, 30]. Our findings offer a concrete mechanism for how KRAS mutations can hijack and potentiate this pathway’s pro-metastatic arm: by upregulating the key type II receptor, thus sensitizing cancer cells to TGF-β ligands in the tumor microenvironment. The striking clinical observation that the ZBTB20-TGFBR2 expression correlation is exclusive to KRAS-mutant metastatic patients strongly supports the idea that the engagement of this axis is a specific evolutionary step taken by these tumors during their progression. This clinical relevance is powerfully corroborated by our survival analysis, which demonstrates that high TGFBR2 expression—the direct outcome of ZBTB20 activation—is a significant predictor of tumor recurrence (Supplemental Figure S4).

Perhaps the most significant implication of our work is the validation of this axis as a druggable vulnerability. We demonstrate that pharmacological degradation of TGFBR2 using ITD-1 is a highly effective anti-metastatic strategy. Critically, its potent efficacy in suppressing metastatic colonization and outgrowth in both liver and lung models suggests that this pathway is not merely required for the initial steps of dissemination but is a sustained dependency for survival and proliferation in diverse organ microenvironments. This broad effectiveness positions TGFBR2 degradation as a robust therapeutic approach [30–33]. However, the strategy of systemically inhibiting TGF-β signaling warrants careful consideration due to its pleiotropic and essential roles in normal physiology. A primary concern, highlighted by clinical trials of systemic TGF-β pathway inhibitors like galunisertib, is the risk of cardiotoxicity, including the development of valvular heart disease [34, 35]. Furthermore, as a master regulator of immune tolerance, abrogating TGF-β signaling presents a double-edged sword. While its inhibition can enhance immune-mediated tumor destruction, as demonstrated in CRC models CRC models [36], it also carries a theoretical risk of disrupting systemic immune homeostasis and inducing autoimmune-like toxicities. Therefore, a critical challenge for future therapeutic development will be to widen the therapeutic window through strategies such as developing tumor-targeted delivery systems, designing next-generation inhibitors with improved safety profiles, or identifying synergistic combinations that could allow for lower, less toxic dosing regimens.

This study has several limitations that represent important areas for future research. First, our conclusions from the tissue microarray analysis are based on a relatively modest sample size, though mitigated by consistency with larger public datasets. Second, our functional studies were intentionally focused on KRAS-mutant cell line models to dissect the clinically observed phenomenon; exploring ZBTB20 function in KRAS-WT models could reveal additional context-dependent roles. Third, we acknowledge that determining the precise subcellular localization of ZBTB20 from TMA sections can be challenging; our core mechanistic conclusions regarding nuclear translocation are primarily derived from our highly controlled in vitro immunofluorescence experiments. Fourth, it should be noted that our in vivo studies utilized experimental metastasis models, which specifically assess the later stages of colonization and outgrowth rather than the entire metastatic cascade. Our focus was also primarily on the cell-autonomous functions of ZBTB20; its potential impact on interactions within the tumor microenvironment was not explored [36]. An important future direction will be to investigate the function of the ZBTB20 axis in the context of CRC’s genetic heterogeneity, particularly in cells harboring mutations in key TGF-β pathway components such as *TGFBR2* or *SMAD4*. Finally, while our findings cumulatively demonstrate that TGFBR2 is a direct transcriptional target, an additional fine-mapping step via ChIP-qPCR would be valuable. Building on this, a comprehensive analysis of the ZBTB20-regulated transcriptome via ZBTB20 ChIP-seq could reveal other nodes in the metastatic network that may also serve as therapeutic targets [23, 37], a strategy that has proven highly effective for defining the regulatory networks of other key metastatic transcription factors in CRC [38].

## Conclusions

In conclusion, our study uncovers ZBTB20 as a critical, phosphorylation-regulated driver of metastasis in *KRAS*-mutant CRC. We provide significant mechanistic insights by delineating the KRAS-ERK-ZBTB20-TGFBR2 pathway and demonstrate that targeting this axis through TGFBR2 degradation is a broadly effective strategy for inhibiting metastasis, offering a promising path forward for treating this aggressive disease.

## Supplementary Information

Supplementary Material 1.

## Data Availability

The datasets used and/or analyzed during the current study are available from the corresponding author on reasonable request.

## Abbreviations

ChIP-seq: Chromatin Immunoprecipitation Sequencing
Co-IP: Co-immunoprecipitation
CPTAC: Clinical Proteomic Tumor Analysis Consortium
CRC: Colorectal Cancer
EMSA: Electrophoretic Mobility Shift Assay
EMT: Epithelial-Mesenchymal Transition
GSEA: Gene Set Enrichment Analysis
H&E: Hematoxylin and Eosin
IHC: Immunohistochemistry
KRAS: Kirsten Rat Sarcoma Viral Oncogene Homolog
LC–MS/MS: Liquid Chromatography-Tandem Mass Spectrometry
MAPK: Mitogen-Activated Protein Kinase
TCGA: The Cancer Genome Atlas
TGFBR2: Transforming Growth Factor Beta Receptor 2
TMA: Tissue Microarray
ZBTB20: Zinc Finger and BTB Domain-Containing Protein 20
