# Supplementary material for: KRAS-ERK signaling drives metastasis in colorectal cancer via phosphorylation-dependent activation of the ZBTB20-TGFBR2 axis
Source: J Exp Clin Cancer Res. 2026 Jan 2;45:29. doi: 10.1186/s13046-025-03619-w (PMC12865998; doi:10.1186/s13046-025-03619-w)
Supplement: Supplementary file 1 — Supplementary Material 1. [file 13046_2025_3619_MOESM1_ESM.pdf]

Supplementary Methods

Cell Lines and Culture

The human colorectal cancer (CRC) cell lines SW480 (KRAS G12V mutant, primary) and SW620 (KRAS G12V mutant, metastatic, isogenic to SW480), along with HEK293T cells, were obtained from the American Type Culture Collection (ATCC, Manassas, VA, USA). All cell lines were authenticated by Short Tandem Repeat (STR) profiling and routinely tested to ensure they were mycoplasma-free. SW480 and SW620 cells were maintained in Leibovitz's L-15 medium supplemented with 10% fetal bovine serum (FBS) at 37°C in a CO_2-free atmosphere. HEK293T cells were cultured in Dulbecco's Modified Eagle Medium (DMEM) supplemented with 10% FBS, 100 U/mL penicillin, and 100 µg/mL streptomycin at 37°C in a humidified 5% CO_2 incubator.

Reagents and Antibodies

The MEK/ERK inhibitor AZD8330 (Selleckchem, S1035) and the TGFBR2 degrader ITD-1 (Selleckchem, S8712) were dissolved in DMSO and used at the indicated concentrations. Recombinant human Epidermal Growth Factor (EGF) was from PeproTech (100-18B).

Primary antibodies used for Western blotting (WB), Immunohistochemistry (IHC), and Immunofluorescence (IF) were: ZBTB20 (Abcam, ab104845), ERK1/2 (Cell Signaling Technology [CST], #4695), phospho-ERK1/2 (Thr202/Tyr204, CST, #4370), phospho-MAPK/CDK Substrates (PXS*P or S*PXR/K) (pTP motif, CST, #2325), TGFBR2 (CST, #79799), E-cadherin (CST, #3195), N-cadherin (CST, #13116), SNAIL (CST, #3879),

ZEB1 (CST, #3396), FLAG-tag (Sigma-Aldrich, F1804), β-actin (Sigma-Aldrich, A5441), GAPDH (CST, #5174), and Lamin B1 (Abcam, ab16048). HRP-conjugated and fluorescent secondary antibodies were from CST or Jackson ImmunoResearch.

Plasmids, RNA Interference, and Stable Cell Line Generation

Human *ZBTB20* cDNA was cloned into pcDNA3.1-FLAG for transient overexpression and into the pLVX-Puro lentiviral vector for stable overexpression. Threonine-to-alanine point mutants (T138A, T142A, T232A) and the triple mutant (3TA) were generated using the Q5 Site-Directed Mutagenesis Kit (NEB, E0554S).

For transient RNA interference, siRNA targeting *TGFBR2* (siTGFBR2) and a non-targeting control siRNA (siNC) were obtained from Dharmacon. For transient plasmid and siRNA transfections, cells were treated with Lipofectamine 3000 (Invitrogen) and Lipofectamine RNAiMAX (Invitrogen), respectively, according to the manufacturer's instructions.

To generate stable cell lines, lentiviral particles were used. For ZBTB20 knockdown, two independent shRNA sequences targeting human *ZBTB20* (shZBTB20) and a non-targeting scramble control (shCtrl) were cloned into the pLKO.1-puro vector. Lentiviral particles were produced in HEK293T cells by co-transfecting the expression vector (pLVX-ZBTB20 or pLKO.1-shZBTB20) with the packaging plasmids psPAX2 and pMD2.G. SW480 or SW620 cells were then transduced with the collected viral supernatants, and stable clones were selected and maintained in medium containing puromycin.

siRNA and Transient Transfection

siRNA targeting TGFBR2 (siTGFBR2) and non-targeting control siRNA (siNC) were from Dharmacon. Plasmids and siRNAs were transfected using Lipofectamine 3000 (Invitrogen) and Lipofectamine RNAiMAX (Invitrogen), respectively, according to the manufacturer's instructions.

RNA Extraction and Quantitative Real-Time PCR (qRT-PCR)

Total RNA was extracted using TRIzol reagent (Invitrogen). cDNA was synthesized using the High-Capacity cDNA Reverse Transcription Kit (Applied Biosystems). qRT-PCR was performed using SYBR Green Master Mix (Applied Biosystems) on a QuantStudio 7 Flex Real-Time PCR System. Relative gene expression was calculated using the $2^{-\Delta\Delta Ct}$ method, normalized to GAPDH.

Western Blotting (WB) and Immunoprecipitation (IP)

Cells were lysed in RIPA buffer supplemented with protease and phosphatase inhibitors (Roche). For nuclear/cytoplasmic fractionation, the NE-PER kit (Thermo Fisher Scientific) was used. Protein concentrations were determined by BCA assay (Thermo Fisher Scientific). Equal amounts of protein were separated by SDS-PAGE, transferred to PVDF membranes (Millipore), and immunoblotted. For IP, cell lysates were incubated with the indicated primary antibody or control IgG overnight, followed by incubation with Protein A/G magnetic beads (Thermo Fisher Scientific). Bound proteins were eluted and analyzed by WB.

Silver Staining and Mass Spectrometry

FLAG-ZBTB20 was immunoprecipitated from SW480 cells and resolved by SDS-PAGE. The gel was stained using a Silver Stain Kit (Thermo Fisher Scientific, 24612).

The protein band corresponding to MAPK1/ERK2 was excised and subjected to in-gel trypsin digestion and subsequent analysis by liquid chromatography-tandem mass spectrometry (LC-MS/MS).

Immunohistochemistry (IHC) and Immunofluorescence (IF)

A human CRC tissue microarray (TMA) was used for IHC. The TMA contained a total of 40 cases, including 15 KRAS-mutant, 20 KRAS-wild-type, and 5 with unknown KRAS status. Formalin-fixed, paraffin-embedded sections were deparaffinized, subjected to antigen retrieval, and incubated with primary antibodies. Detection was performed using a HRP-polymer system (Dako) and DAB substrate. Following staining and quality control inspection, cases with significant tissue loss or artifacts were excluded from the final analysis. This resulted in a final cohort of 13 KRAS-mutant and 16 KRAS-wild-type patient samples for quantification, which were used for all TMA-based analyses in this study. An H-score (intensity × percentage) was calculated. For IF, cells grown on coverslips were fixed, permeabilized, blocked, and incubated with primary and fluorescent secondary antibodies. Nuclei were counterstained with DAPI. Images were captured using a fluorescence microscope.

Migration and Invasion Assays

Cell migration and invasion were assessed using Transwell chambers (8 μm pore size, Corning) without (migration) or with (invasion) Matrigel coating (Corning). Cells were seeded in the upper chamber in serum-free medium, with 10% FBS medium in the lower chamber as a chemoattractant. After 24-48 hours, cells on the lower surface were fixed, stained with crystal violet, and counted.

Animal Studies

All animal experiments were approved by the Institutional Animal Care and Use Committee. 6-8 week old male BALB/c nude mice were used. Metastatic burden was monitored by bioluminescence imaging (BLI) using an IVIS Spectrum system (PerkinElmer).

- **ZBTB20 Loss-of-Function Model:** SW620-scramble or SW620-shZBTB20 cells (3×10^6) were injected into the left lobe of the liver, followed by immediate splenectomy to model liver metastasis.
- **ZBTB20 Phosphorylation-Mutant Models:** SW480-Vector, SW480-WT ZBTB20, or SW480-3T/A ZBTB20 cells were used. For the intrahepatic model, 3×10^6 cells were injected into a liver lobe. For the hematogenous model, 3×10^6 cells were injected via the lateral tail vein.
- **Pharmacological Inhibition Model:** SW620 cells (3×10^6) were used. For liver colonization, cells were injected intrahepatically. For lung colonization, cells were injected via the tail vein. Treatment with ITD-1 (e.g., 20 mg/kg, daily, intraperitoneal injection) or vehicle (PBS) was initiated on day 3 post-injection. At the endpoint, organs were harvested for ex vivo imaging, H&E staining, and IHC.

RNA Sequencing and Bioinformatic Analysis

Total RNA was extracted from SW620-shCtrl and SW620-shZBTB20 cells (n=2 biological replicates per group) and subjected to library preparation and sequencing on an Illumina NovaSeq platform. Raw reads were aligned to the human genome (hg38), and differential gene expression was analyzed using the DESeq2 package in R. Gene

Set Enrichment Analysis (GSEA) was performed using the GSEA software (Broad Institute) to identify enriched hallmark gene sets. For cistromic analysis, publicly available ZBTB20 ChIP-seq data from the ENCODE Project were interrogated for binding peaks within the promoter regions (-2kb to +1kb of the transcriptional start site, TSS) of the differentially expressed genes identified in our RNA-seq. Clinical data were analyzed to determine the expression of *ZBTB20*, its correlation with KRAS status and metastatic stage, and its correlation with *TGFBR2* expression. This was performed using data from The Cancer Genome Atlas (TCGA) COAD/READ cohort and the CPTAC Colon Cancer dataset. Patient survival analysis was conducted using the GEPIA2 web server. Additionally, publicly available Gene Expression Omnibus (GEO) datasets (GSE89524, GSE130236) were analyzed to compare *ZBTB20* expression between SW480 and SW620 cell lines.

Luciferase Reporter Assay

HEK293T cells were co-transfected with a pGL3-TGFBR2 promoter construct, a ZBTB20 expression vector, and a Renilla luciferase vector (pRL-TK, Promela) for normalization. Luciferase activity was measured 48 hours later using the Dual-Luciferase Reporter Assay System (Promega).

TUNEL Assay

SW620 cells (parental or stable knockdown lines) were seeded into confocal dishes at a density of 5×10^5 cells/dish and cultured overnight. After washing with PBS, cells were fixed with 4% paraformaldehyde for 30 minutes at room temperature, followed by three washes with PBS. Cells were then permeabilized with 0.3% Triton X-100 in

PBS for 5 minutes. Apoptosis was detected using the One Step TUNEL Apoptosis Assay Kit (Beyotime, C1086) according to the manufacturer's protocol. Briefly, cells were incubated with 50 μL of TUNEL reaction buffer for 60 minutes at 37°C in the dark. Nuclei were subsequently counterstained with DAPI for 15 minutes. Images were captured using a fluorescence microscope.

Electrophoretic Mobility Shift Assay (EMSA)

Biotin-labeled DNA probes for the *TGFBR2* promoter were incubated with recombinant human ZBTB20 protein (purified in house). For competition and supershift assays, unlabeled specific probes or anti-ZBTB20 antibody were added. Protein-DNA complexes were resolved on native polyacrylamide gels and detected by streptavidin-HRP and chemiluminescence.

Statistical Analysis

Data are presented as mean ± standard deviation (SD) from at least three independent experiments unless otherwise stated. Statistical significance was determined using a two-tailed Student's t-test (two groups) or one-way ANOVA with post-hoc tests (multiple groups) using GraphPad Prism software. Survival analysis was performed using Kaplan-Meier curves and log-rank tests. Correlations were assessed using Pearson correlation coefficients. A p-value < 0.05 was considered statistically significant (*$p < 0.05$, **$p < 0.01$, ***$p < 0.001$, ****$p < 0.0001$).

Supplementary Figure Legends:

Supplemental Figure S1. ZBTB20 Does Not Affect Colorectal Cancer Cell Proliferation *In Vitro*.

(A,B) CCK-8 proliferation assays of SW620 cells with ZBTB20 knockdown (A) and SW480 cells with ZBTB20 overexpression (B). Data are presented as mean ± SD. (C,D) Colony formation assays and corresponding quantification for SW620 cells with ZBTB20 knockdown (C) and SW480 cells with ZBTB20 overexpression (D). Data are presented as mean ± SD. Statistical significance was determined by one-way ANOVA. ns, not significant.

Supplemental Figure S2. ERK-Mediated Phosphorylation Governs ZBTB20's Nuclear Localization and Pro-Metastatic Activity.

(A) Immunofluorescence and quantification of ZBTB20 subcellular localization in HEK293T cells expressing either WT ZBTB20 or the ZBTB20-3T/A mutant. Cells were serum-starved and treated as indicated (EGF, 10 ng/mL; AZD8330, 10 μM). Green: ZBTB20; Blue: DAPI (nuclei). (B) ZBTB20 phosphorylation is essential for its pro-metastatic activity in a lung colonization model. SW480 stable cell lines (Vector, WT ZBTB20, or ZBTB20-3T/A; 3×10^6 cells per mouse) were injected via the tail vein into male BALB/c nude mice (n=5 mice per group). Representative images of whole lungs, H&E sections, and immunohistochemical staining for ZBTB20 and TGFBR2 in metastatic nodules are shown. Quantification of lung tumor area is presented in the bar graph.

Data in the bar graph are presented as mean ± SD. *$p < 0.05$. Statistical analysis was performed using one-way ANOVA. Scale bars are as indicated.

Supplemental Figure S3. Bioinformatic analysis of ZBTB20 downstream targets.

(A) Heatmap of differentially expressed genes from RNA-seq analysis of SW620 cells with ZBTB20 knockdown versus scramble control. **(B)** KEGG pathway enrichment analysis of all differentially expressed genes upon ZBTB20 knockdown. **(C)** Heatmaps of selected genes from the GSEA analysis, showing downregulation of genes in the EMT (left) and TGF-β (right) pathways following ZBTB20 knockdown. **(D)** Pie chart illustrating the genomic distribution of ZBTB20 binding peaks from ChIP-seq data, showing a majority of peaks located in promoter regions.

Supplemental Figure S4. Prognostic Significance of TGFBR2 Expression in Colorectal Cancer.

Kaplan-Meier analyses of patient survival from the TCGA CRC cohort, stratified by high vs. low TGFBR2 expression. (A) High TGFBR2 expression is associated with a trend towards poorer Overall Survival (OS) ($p=0.09$). (B) High TGFBR2 expression is significantly correlated with worse Disease-Free Survival (DFS) ($p=0.0063$). P-values were determined by the log-rank test. The Y-axis label "Percent survival" has been updated to the more standard "Cumulative survival" or "Survival probability" as per best practice.

Supplemental Figure S5. Knockdown of ZBTB20 or TGFBR2 does not induce apoptosis in KRAS-mutant CRC cells.

(A) Western blot analysis of total and cleaved caspase-3, and ZBTB20 expression in SW620 cells stably transfected with control shRNA (Control) or two independent shRNAs targeting ZBTB20 (ZBTB20-Sh1, ZBTB20-Sh2). GAPDH served as a loading control.

(B) Representative fluorescence microscopy images (upper) and corresponding quantification (bottom) of TUNEL assays in the SW620 cell lines described in (A). Green fluorescence indicates TUNEL-positive apoptotic cells; blue fluorescence (DAPI) indicates cell nuclei. Scale bar, 50μm.

(C) Western blot analysis of total and cleaved caspase-3, and TGFBR2 expression in SW620 cells transfected with non-targeting control siRNA (Control) or two independent siRNAs targeting TGFBR2 (TGFBR2-siRNA-1, TGFBR2-siRNA-2).

(D) Representative fluorescence microscopy images (upper) and corresponding quantification (bottom) of TUNEL assays in SW620 cells treated as described in (C).

Data in bar graphs are presented as mean ± SD from three independent experiments. Statistical significance was determined by one-way ANOVA. ns, not significant.

Figure S1

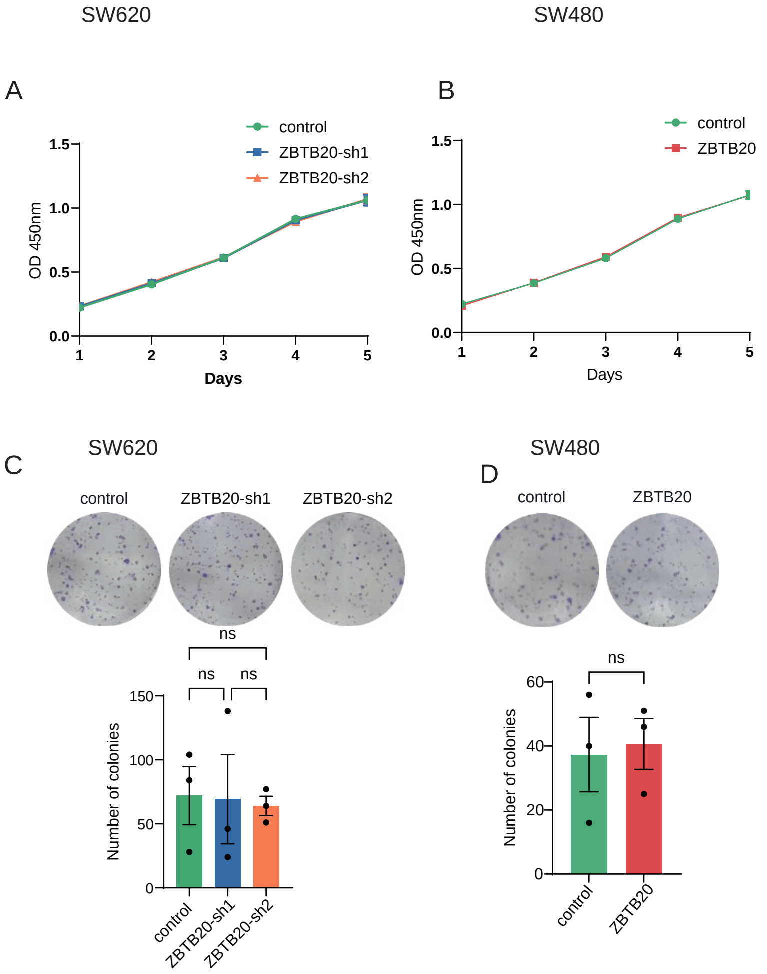

SW620
SW480
A
control
ZBTB20-sh1
ZBTB20-sh2
OD 450nm
1.5
1.0
0.5
0.0
1
2
3
4
5
Days
B
control
ZBTB20
OD 450nm
1.5
1.0
0.5
0.0
1
2
3
4
5
Days
SW620
C
control
ZBTB20-sh1
ZBTB20-sh2
ns
ns
ns
150
100
50
0
Number of colonies
control
ZBTB20-sh1
ZBTB20-sh2
SW480
D
control
ZBTB20
ns
60
40
20
0
Number of colonies
control
ZBTB20

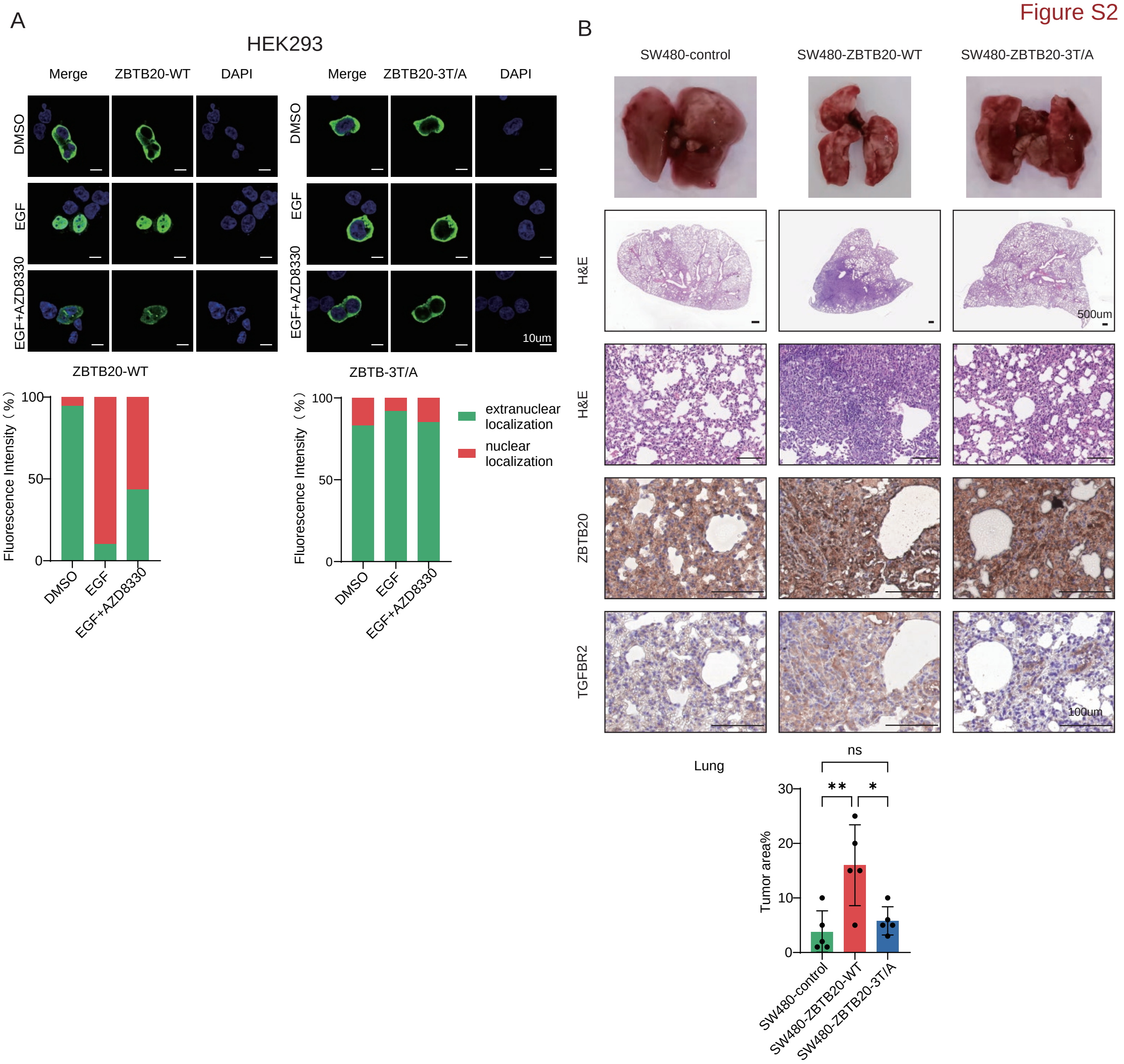

Figure S2
A
HEK293
Merge
ZBTB20-WT
DAPI
Merge
ZBTB20-3T/A
DAPI
DMSO
EGF
EGF+AZD8330
10um
ZBTB20-WT
ZBTB-3T/A
Fluorescence Intensity（%）
100
50
0
DMSO
EGF
EGF+AZD8330
extranuclear localization
nuclear localization
B
SW480-control
SW480-ZBTB20-WT
SW480-ZBTB20-3T/A
H&E
ZBTB20
TGFBR2
500um
100um
Lung
ns
**
*
Tumor area%
30
20
10
0

Figure S3

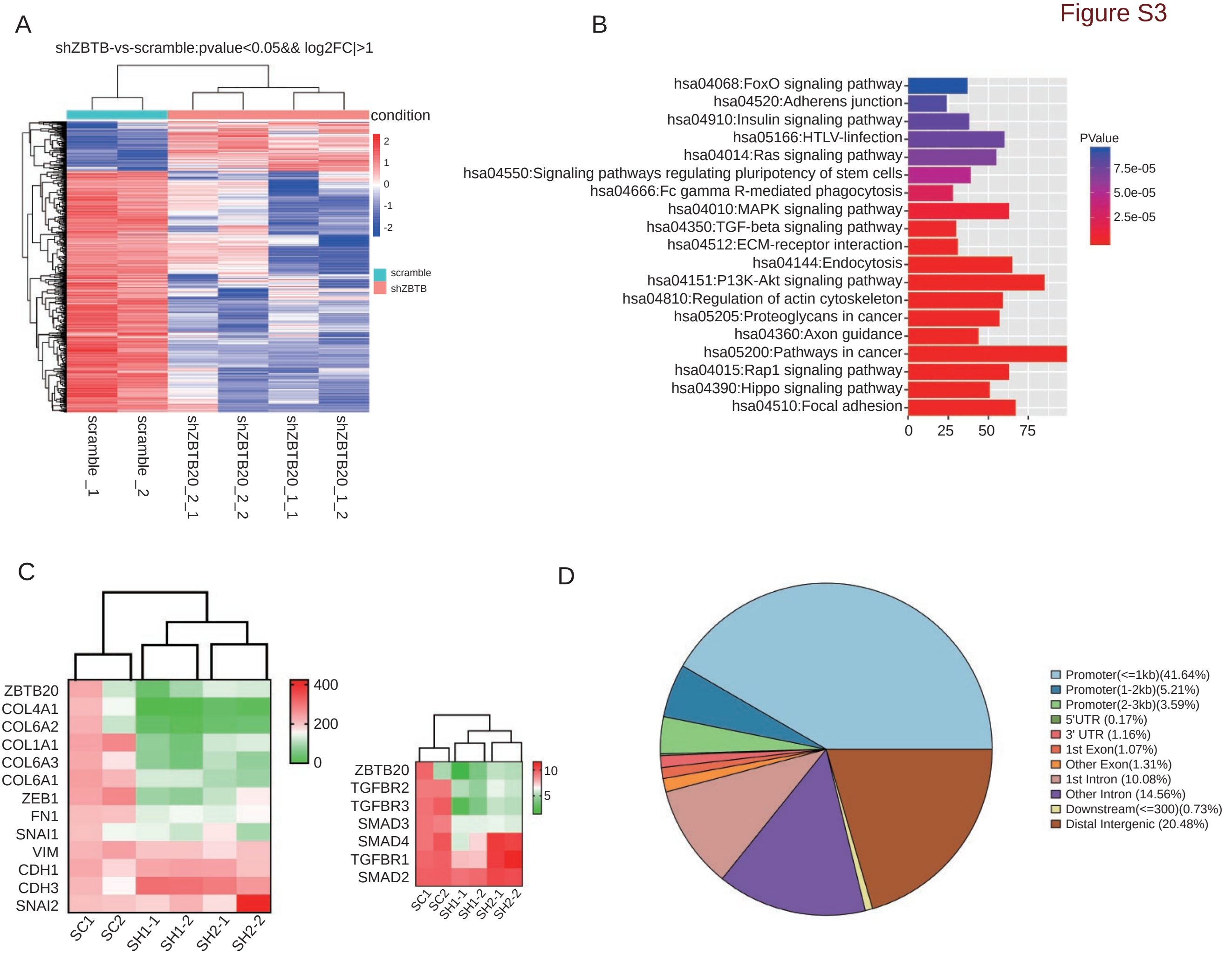
A
shZBTB-vs-scramble:pvalue<0.05&& log2FC|>1
condition
2
1
0
-1
-2
scramble
shZBTB
scramble_1
scramble_2
shZBTB20_2_1
shZBTB20_2_2
shZBTB20_1_1
shZBTB20_1_2
B
hsa04068:FoxO signaling pathway
hsa04520:Adherens junction
hsa04910:Insulin signaling pathway
hsa05166:HTLV-linfection
hsa04014:Ras signaling pathway
hsa04550:Signaling pathways regulating pluripotency of stem cells
hsa04666:Fc gamma R-mediated phagocytosis
hsa04010:MAPK signaling pathway
hsa04350:TGF-beta signaling pathway
hsa04512:ECM-receptor interaction
hsa04144:Endocytosis
hsa04151:P13K-Akt signaling pathway
hsa04810:Regulation of actin cytoskeleton
hsa05205:Proteoglycans in cancer
hsa04360:Axon guidance
hsa05200:Pathways in cancer
hsa04015:Rap1 signaling pathway
hsa04390:Hippo signaling pathway
hsa04510:Focal adhesion
0
25
50
75
PValue
7.5e-05
5.0e-05
2.5e-05
C
ZBTB20
COL4A1
COL6A2
COL1A1
COL6A3
COL6A1
ZEB1
FN1
SNAI1
VIM
CDH1
CDH3
SNAI2
SC1
SC2
SH1-1
SH1-2
SH2-1
SH2-2
400
200
0
ZBTB20
TGFBR2
TGFBR3
SMAD3
SMAD4
TGFBR1
SMAD2
SC1
SC2
SH1-1
SH1-2
SH2-1
SH2-2
10
5
D
Promoter(<=1kb)(41.64%)
Promoter(1-2kb)(5.21%)
Promoter(2-3kb)(3.59%)
5'UTR (0.17%)
3' UTR (1.16%)
1st Exon(1.07%)
Other Exon(1.31%)
1st Intron (10.08%)
Other Intron (14.56%)
Downstream(<=300)(0.73%)
Distal Intergenic (20.48%)

Figure S4

A

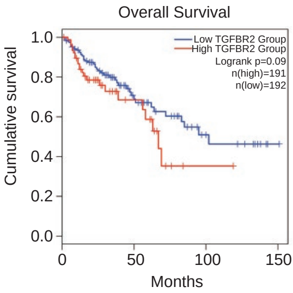
Overall Survival
Cumulative survival
1.0
0.8
0.6
0.4
0.2
0.0
0
50
100
150
Months
Low TGFBR2 Group
High TGFBR2 Group
Logrank p=0.09
n(high)=191
n(low)=192

B

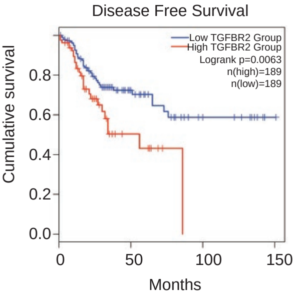
Disease Free Survival
Cumulative survival
0.8
0.6
0.4
0.2
0.0
0
50
100
150
Months
Low TGFBR2 Group
High TGFBR2 Group
Logrank p=0.0063
n(high)=189
n(low)=189

Figure S5

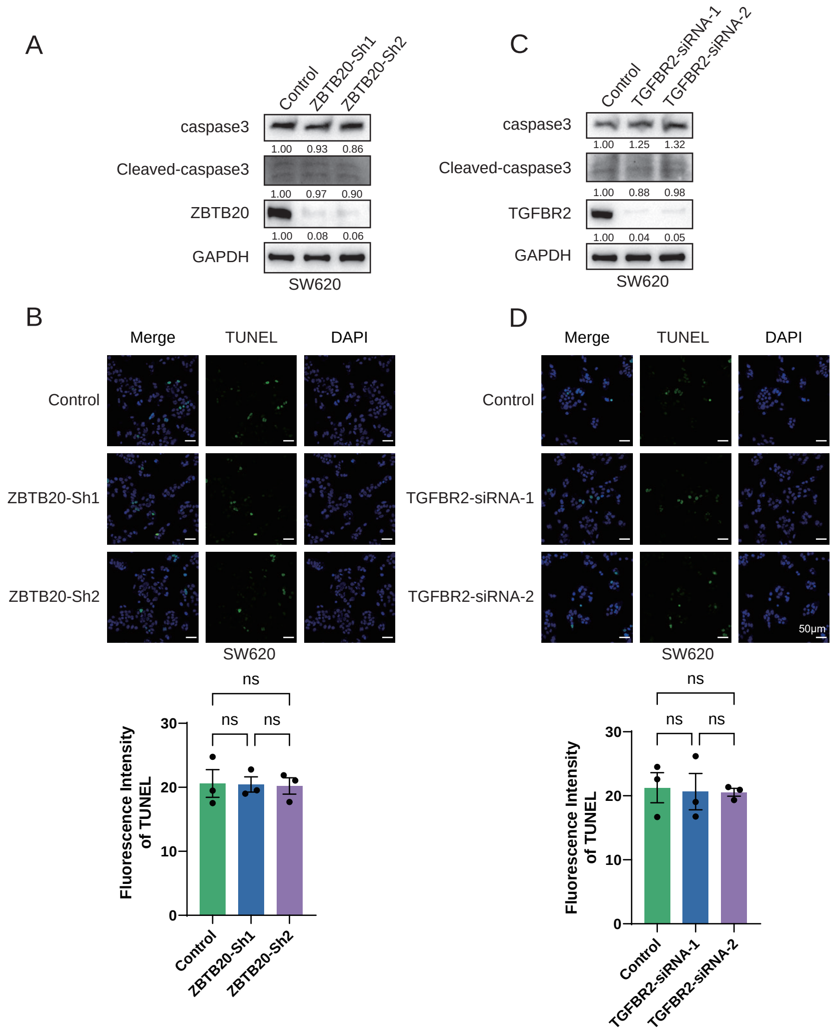
A
Control
ZBTB20-Sh1
ZBTB20-Sh2
caspase3
1.00 0.93 0.86
Cleaved-caspase3
1.00 0.97 0.90
ZBTB20
1.00 0.08 0.06
GAPDH
SW620
C
Control
TGFBR2-siRNA-1
TGFBR2-siRNA-2
caspase3
1.00 1.25 1.32
Cleaved-caspase3
1.00 0.88 0.98
TGFBR2
1.00 0.04 0.05
GAPDH
SW620
B
Merge
TUNEL
DAPI
Control
ZBTB20-Sh1
ZBTB20-Sh2
SW620
D
Merge
TUNEL
DAPI
Control
TGFBR2-siRNA-1
TGFBR2-siRNA-2
50μm
SW620
ns
ns
ns
Fluorescence Intensity of TUNEL
30
20
10
0
Control
ZBTB20-Sh1
ZBTB20-Sh2
ns
ns
ns
Fluorescence Intensity of TUNEL
30
20
10
0
Control
TGFBR2-siRNA-1
TGFBR2-siRNA-2
